# Fostering adolescent engagement in generative AI art therapy: a dual SEM-ANN analysis of emotional

**DOI:** 10.3389/fpsyg.2025.1628471

**Published:** 2025-07-23

**Authors:** Xiaojie Peng, Yanran Qian, Qian Bao

**Affiliations:** Department of Design, Graduate School, Hanyang University, Seoul, Republic of Korea

**Keywords:** generative AI painting, digital art therapy, interest-driven, emotional design theory, artificial neural network (ANN), TAM model

## Abstract

**Introduction:**

This study explores the application of generative artificial intelligence (AI) art in digital art therapy, focusing on how it influences adolescents’ interest-driven participation. With mental health concerns rising among youth, understanding motivational mechanisms in AI-assisted therapeutic tools is both timely and essential.

**Methods:**

A cross-sectional survey was conducted with 444 junior and senior high school students in Hubei Province, China. The study integrated Emotional Design Theory and the Technology Acceptance Model (TAM) to construct a predictive model. Structural equation modeling (SEM) and artificial neural network (ANN) analyses were employed to validate the model and identify key predictors of engagement.

**Results:**

SEM results indicated that perceived usefulness (PU), perceived ease of use (PEOU), perceived fun (PF), and perceived trust (PT) significantly influenced users’ attitudes toward use (ATT) (*p* < 0.001). ATT, PF, and PT were strong predictors of interest-driven participation, while the behavioral level had no direct effect on perceived enjoyment (PE). ANN analysis further highlighted ATT as the most influential predictor (100% normalized importance), notably exceeding PE (19.3%).

**Discussion:**

These findings emphasize the importance of intuitive design, seamless interaction, and trust-building in sustaining adolescents’ engagement with AI-based art therapy. The study provides a theoretical foundation for understanding interest formation in youth and offers practical implications for improving emotional design, digital therapeutic tools, and mental health interventions.

## 1 Introduction

With the rapid advancement of artificial intelligence (AI) technologies in education, mental health, and artistic creation, the investigation of adolescents’ engagement with AI tools has become an increasingly urgent research priority (Bommasani et al., 2021). Adolescence is recognized as a critical developmental stage, marked by the accelerated formation of self-identity and the maturation of emotional expressiveness (Polanczyk et al., 2015). The timely introduction of appropriate intervention tools during this stage is considered essential for fostering psychological resilience and expressive capabilities. Concurrently, generative AI-based painting technologies have been widely adopted in the fields of digital art and psychological intervention, owing to their strengths in automated creation and multimodal interaction (Jütte et al., 2024).

Recently, generative artificial intelligence (AI) tools have been increasingly integrated into routine psychotherapeutic practices (Yap et al., 2024). Growing evidence has suggested that this technology provides distinct advantages in remote delivery (Arnskötter et al., 2024), privacy protection (Du et al., 2024), and diversified visual generation (Ho and Lee, 2023), rendering it particularly well-suited to addressing adolescents’ rising demand for interactivity and personalization in the digital era (Zubala et al., 2021). Moreover, generative AI has been shown to exert positive effects on emotional resonance, self-expression, and psychological comfort, and is now widely regarded as an innovative approach in the context of mental health interventions (Cuijpers et al., 2016; Du et al., 2024; World Health Organization, 2015).

Although prior studies have provided preliminary evidence regarding the potential of generative AI in art-based therapy, the existing literature has primarily concentrated on short-term changes in user satisfaction and engagement. Comparatively little attention has been devoted to the underlying mechanisms of interest formation and their long-term impact on the sustainability of therapeutic interventions (Marker et al., 2013). Previous findings (e.g., Marker et al., 2013) have demonstrated that perceived fun, enjoyment, and trust significantly influence users’ acceptance and engagement with AI-based art tools (Blanco et al., 2010; Ho and Lee, 2023; Yap et al., 2024). However, in the context of adolescent psychological intervention, systematic empirical investigations remain limited concerning how these perceptual factors are converted into sustained interest and long-term usage motivation—an issue that warrants further scholarly attention. Given that interest and motivation have been widely acknowledged as critical determinants of intervention adherence and therapeutic effectiveness among adolescents (Sheppard et al., 2018), the present study seeks to examine the underlying pathways of interest-driven mechanisms in the use of generative AI painting tools. In doing so, this research aims to bridge the existing gap between theoretical understanding and applied practice within the field.

To address the limitations, this study proposes an integrated model that combines Emotional Design Theory (EDT) (Norman, 2007) and the Technology Acceptance Model (TAM) (Davis, 1989) to explain the mechanisms underlying interest-driven engagement among adolescents. By employing a combined approach of Structural Equation Modeling (SEM) and Artificial Neural Networks (ANN), the study aims to analyze the relationships among perception, emotional responses, and behavioral tendencies, and to validate the predictive effects of these variables on sustained interest in continued use.

This study is designed to identify the key factors that influence adolescents’ sustained engagement with generative AI-based painting tools and to provide both theoretical and empirical support for AI-assisted mental health interventions. Accordingly, the following three research questions are formulated:

RQ1: To what extent are Perceived Usefulness (PU), Perceived Ease of Use (PEOU), and Attitude toward Use (ATT), as defined within the Technology Acceptance Model (TAM), associated with adolescents’ interest in generative AI tools?
RQ2: Based on Emotional Design Theory (EDT), in what ways do generative AI tools used in therapeutic contexts elicit emotional responses at the visceral, behavioral, and reflective levels among adolescents?
RQ3: To what extent do Perceived Enjoyment (PE), Perceived Fun (PF), and Perceived Trust (PT) influence adolescents perceived attractiveness toward AI-based painting tools?

This study not only extends the application of Emotional Design Theory (EDT) and the Technology Acceptance Model (TAM) within the field of art-based therapy but also provides actionable insights for interface design and educational practices in AI-assisted psychological interventions. By identifying the key pathways influencing adolescents’ interest in generative AI tools, the findings are expected to support the refinement of intervention strategies by educators and therapists, while also offering design-oriented guidance to developers of AI-based painting platforms for enhancing user engagement and fostering trust.

## 2 Literature review and hypothesis development

### 2.1 Digital art therapy

Digital art therapy is an innovative form of psychological intervention that integrates traditional expressive art modalities (e.g., drawing, sculpture) with digital media, including tablets, virtual platforms, and interactive creative tools. In recent years, it has received growing scholarly attention within the field of adolescent mental health (Deaver, 2002; Gazit et al., 2021; Malchiodi, 2011). Grounded in digital technologies, this approach offers a flexible and secure environment for psychological expression through multisensory stimulation, remote accessibility, and personalized interaction—features particularly well aligned with the preferences of the “digital native” generation (Seko et al., 2014; Hartanto et al., 2025).

The therapeutic potential of digital art therapy has been demonstrated in a range of studies, showing its effectiveness in facilitating emotional release, supporting trauma recovery, and promoting cognitive restructuring. Its efficacy has been especially evident in contexts such as school-based mental health programs and the management of developmental disorders (An and Li, 2024; Kapitan, 2009; Saladino, 2023). However, several studies have noted that digital interventions may reduce the sense of immersion and emotional connection fostered by tactile interaction—an element widely regarded as an irreplaceable therapeutic mechanism in traditional face-to-face art therapy (Honchar, 2023). The physical engagement inherent in hands-on artistic creation has been believed to foster deep emotional resonance, which digital formats may fail to replicate effectively. Therefore, digital and traditional art therapy modalities are considered to have distinct advantages and limitations. The former is recognized for its innovative potential in media diversity and intervention accessibility, whereas the latter retains the experiential depth essential for embodied emotional connection.

Although digital art therapy has demonstrated promising applications, existing studies have primarily focused on feasibility, user satisfaction, and short-term engagement. However, a notable gap remains in the literature concerning long-term psychological effects, emotion regulation pathways, and sustained usage patterns. In particular, as emerging technologies such as generative artificial intelligence (AI) are increasingly embedded into digital creative tools, interactions among emotional responses, reflective processes, trust development, and motivational engagement have yet to be systematically modeled or empirically validated. Consequently, there is a pressing need to further investigate the intervention mechanisms and sustained motivational dynamics of generative AI within digital art therapy contexts.

### 2.2 Generative AI painting

Generative artificial intelligence (AI) painting refers to the creation of visual artworks through advanced machine learning models, such as diffusion networks and Generative Adversarial Networks (GANs) (Bandi et al., 2023; Ramesh et al., 2022; Rapp et al., 2025). Since 2020, the rapid advancement of AI technologies has sparked growing interest in both academic and clinical fields regarding the potential applications of such tools (e.g., DALL·E, Midjourney) in mental health interventions (Bommasani et al., 2021). These tools generate images based on natural language prompts and are characterized by a high degree of personalization, contextual relevance, and visual diversity. They are capable of eliciting creative associations and emotional responses in real time, aligning particularly well with adolescents’ psychological expectations for immersive and interactive experiences.

Preliminary efforts have been made to incorporate generative AI tools into digital art therapy workflows. Initial findings suggest that the customized visual content produced by these tools exhibits therapeutic potential in facilitating emotional expression and providing psychological comfort (Ho and Lee, 2023; Kaimal et al., 2016). Moreover, generative AI significantly lowers the technical barriers to artistic creation, offering adolescents—especially those without formal training—a more accessible, visually intuitive, and immediate mode of creative engagement. This, in turn, fosters greater participation and supports the development of aesthetic self-construction (Orr, 2015).

While current studies have confirmed the novelty appeal and positive short-term feedback of generative AI tools, the medium- to long-term psychological mechanisms through which these tools influence emotional regulation, cognitive restructuring, and behavioral transformation remain underexplored. In particular, key psychological variables—such as agency perception, creative ownership, and technological trust—have yet to be systematically incorporated into and empirically validated within theoretical intervention frameworks.

Therefore, a systematic investigation of the psychological intervention mechanisms of generative AI in digital art therapy is warranted. Specifically, clarifying its role in shaping adolescents’ emotional motivations and sustaining continued usage intentions holds significant implications for both theoretical model development and the practical implementation of AI-assisted therapeutic practices.

### 2.3 Emotional Design Theory (EDT) and Technology Acceptance Model (TAM)

Emotional Design Theory (Norman, 2007) proposes that user experience unfolds across three hierarchical levels: the visceral layer, which pertains to immediate sensory impressions and aesthetic gratification; the behavioral layer, which emphasizes functional usability and interactional efficiency; and the reflective layer, which encompasses cognitive appraisal, meaning making, and identity projection (Desmet and Hekkert, 2007). Emotional responses triggered and regulated during user–system interactions are framed within EDT as fundamental psychological drivers of long-term engagement.

Concurrently, the Technology Acceptance Model (TAM) has been widely adopted as a foundational framework for understanding technology adoption behaviors. It centers on two principal constructs: Perceived Usefulness (PU)—the extent to which a technology is believed to enhance task performance—and Perceived Ease of Use (PEOU)—the perceived effort required to learn and operate the system (Holden and Karsh, 2010; Radner and Rothschild, 1975). TAM has been extensively validated in domains such as education, healthcare, and information systems, and has recently demonstrated high explanatory power in predicting adolescents’ acceptance of AI-enhanced educational technologies (Adams et al., 1992; Hu et al., 1999).

For example, Soliman et al. (2024) applied a hybrid Partial Least Squares Structural Equation Modeling (PLS-SEM) and Artificial Neural Network (ANN) approach to examine the sustained use of generative AI tools among university students. Their findings highlighted the importance of variables such as PU, trust, and autonomy. In a subsequent 2025 study, fuzzy-set Qualitative Comparative Analysis (fsQCA) revealed that a combined pathway of perceived enjoyment and trust emerged as a core configuration predicting high adoption behavior (Soliman et al., 2024, 2025). These results support the integration of multimethod analytical techniques and emphasize the interplay between cognitive and affective constructs in user engagement.

While other behavioral theories—such as the Theory of Planned Behavior (TPB), the Unified Theory of Acceptance and Use of Technology (UTAUT), and Diffusion of Innovations (DOI)—have also been utilized to explain digital intervention adoption, they predominantly emphasize social, normative, or institutional variables. As such, these frameworks often overlook the aesthetic and emotional factors crucial in contexts like art therapy. To address this limitation, the present study integrates TAM’s cognitive appraisal pathway with EDT’s affective experience pathway, with the aim of systematically elucidating the cognitive–affective–behavioral mechanisms that underpin adolescents’ engagement with generative AI-based painting tools.

To avoid conceptual overlap, three affective constructs were further distinguished according to motivational orientation and emotional valence:

Perceived Fun (PF): An extrinsically motivated construct reflecting stimulation from novelty and playfulness.
Perceived Enjoyment (PE): An intrinsically driven affective state derived from the inherent pleasure of the activity itself.
Perceived Trust (PT): A user’s confidence in the AI tool’s reliability, privacy protection, and response accuracy.

In summary, TAM offers a robust foundation for cognitive evaluation, while EDT complements this by capturing the emotional dynamics that shape behavioral intention. The integration of these two models enables the construction of a more comprehensive and multidimensional theoretical framework. This synthesis is consistent with emerging scholarship on the pedagogical and therapeutic potential of generative AI (Ali et al., 2025; Soliman et al., 2024) and provides a conceptual foundation for the user-centered design of digital art therapy tools tailored to adolescent users.

By addressing both technological and emotional dimensions, this integrative approach facilitates a more nuanced understanding of motivational drivers in adolescent engagement with AI-mediated artistic interventions. Ultimately, the study seeks to illuminate the dynamic interplay among cognition, emotion, and behavior—while filling key theoretical gaps related to dimensional structure and psychological depth in current literature.

### 2.4 Perceived fun (PF), perceived enjoyment (PE), perceived trust (PT)

Perceived fun (PF) is generally defined as the emotional stimulation experienced by users during interaction with a technological product, characterized by feelings of excitement, novelty, and entertainment. Such responses are primarily elicited by external interactive elements—such as dynamic feedback and visual effects—that provoke immediate affective reactions (Venkatesh and Davis, 2000). In contrast, Perceived Enjoyment (PE) refers to the intrinsic satisfaction derived from the activity itself. It reflects a deeper state of immersion and has been consistently associated with long-term usage intentions in empirical research (Gefen et al., 2003; Huang and Liu, 2024). Additionally, Perceived Trust (PT) captures the user’s confidence in the system’s reliability, ethical data handling, and protection of personal privacy—dimensions that are particularly vital in sensitive applications such as psychological interventions (Rahwan, 2018; Sica and Sætra, 2023; Yap et al., 2024).

While these affective constructs have been extensively utilized in the context of artificial intelligence systems (e.g., Van der Heijden, 2004; Gefen et al., 2003), their conceptual definitions and boundaries remain inconsistent across disciplines. For example, Moon and Kim (2001) argued that PF is primarily linked to extrinsic motivation driven by short-term entertainment, whereas PE is associated with intrinsic motivation rooted in sustained engagement. However, in many studies, the two constructs have been used interchangeably without clear structural differentiation, thereby weakening the explanatory capacity of the theoretical frameworks in which they are embedded. This ambiguity is particularly problematic among adolescent users, for whom PF and PE represent distinct motivational pathways—namely, interest initiation and sustained engagement, respectively. These distinctions highlight the need for careful conceptual separation in theoretical modeling.

Meanwhile, the importance of PT has become increasingly evident in the domain of generative AI applications. Soliman et al. (2024) demonstrated that, among university students, PT exhibited a stronger predictive effect on continued usage intention than traditional TAM variables such as Perceived Usefulness (PU) and Perceived Ease of Use (PEOU). In their study, PT significantly influenced user attitudes and was positively associated with both self-disclosure and perceived psychological safety. Further, through fuzzy-set Qualitative Comparative Analysis (fsQCA), Soliman et al. (2025) identified the co-occurrence of high PE and high PT as a core configuration predicting high adoption behavior—underscoring the synergistic effect of emotional variables in technology acceptance pathways.

These insights suggest that, especially in highly personalized AI-assisted art therapy contexts, the relationships among PF, PE, and PT should not be modeled as linear or isolated effects. Instead, their interactive and moderating roles must be explicitly accounted for. Although affective perceptions have been incorporated into extended TAM models, most existing studies have not yet developed robust theoretical structures or provided empirical validations that reflect the developmental particularities of adolescent users and the situational features of AI-mediated psychological interventions.

### 2.5 Research gaps and the necessity of theoretical integration

Despite the increasing academic interest in digital art therapy, generative artificial intelligence (AI), and adolescent psychological interventions, several critical theoretical gaps and practical limitations persist.

First, models that account for interest-driven engagement mechanisms remain largely underdeveloped. Prior research has predominantly concentrated on surface-level factors—such as user satisfaction, interface preferences, and perceived usability—while neglecting the deeper motivational dynamics that support sustained user involvement. In particular, the behavioral transition from initial curiosity to long-term engagement has neither been sufficiently theorized nor empirically validated.

Second, the conceptualization of affective variables lacks consistency across domains. Constructs such as Perceived Fun (PF), Perceived Enjoyment (PE), and Perceived Trust (PT) are variably defined in educational technology, medical AI, and entertainment research, resulting in limited model transferability and reduced cross-contextual applicability. This definitional ambiguity hinders theoretical convergence and weakens the explanatory coherence of existing models.

Third, there is an absence of integrated structural models that unify emotional experience theories with cognitive technology acceptance frameworks. To date, no systematic effort has successfully combined Norman’s Emotional Design Theory (EDT) with the Technology Acceptance Model (TAM) to formulate a comprehensive framework capable of capturing both the affective depth of user experience and the cognitive evaluations that inform technology adoption.

To bridge these gaps, the present study proposes an integrated emotion–cognition–behavioral mechanism model by synthesizing EDT and TAM. This model is designed to elucidate the motivational factors underlying adolescents’ engagement with generative AI-based painting tools in therapeutic contexts. To empirically validate the proposed framework, Structural Equation Modeling (SEM) and Artificial Neural Network (ANN) analyses will be employed to examine the interactive and multidimensional relationships among PF, PE, and PT. This integrative approach is expected to enrich the theoretical foundation and enhance the structural sophistication of research in AI-mediated psychological intervention.

### 2.6 Research hypotheses

#### 2.6.1 Emotional design theory (EDT)

It has been demonstrated by Chang and Chen that the enhancement of positive sensory experience at the instinctive level (IL) of e-textbooks has been shown to elicit students’ positive emotional responses, thereby reducing cognitive load and facilitating improvements in both learning efficiency and academic performance at the behavioral level (BL) (Chang and Chen, 2024). These findings suggest that the instinctive level exerts a significant positive influence on the behavioral level. Similarly, according to Graphics for Learning: Proven Guidelines for Planning, Designing, and Evaluating Visuals in Training Materials, the enhancement of the visual and sensory appeal of instructional materials at the instinctive level (IL) has been found to significantly increase learners’ positive emotional responses, reduce cognitive load, thereby improving both learning efficiency and academic performance at the behavioral level (BL) (Clark and Lyons, 2010). It has been suggested by other studies that, although emotional interest can be enhanced through the integration of engaging textual and visual elements, such modifications do not consistently result in improved learning outcomes. They may, in fact, lead to reduced academic performance as a consequence of heightened cognitive load. A careful balance between emotional and cognitive elements is therefore essential when designing instructional materials to achieve optimal learning outcomes (Harp and Mayer, 1997). Hassenzahl and Tractinsky (2006)showed that optimized behavioral-level design enhances overall product evaluations and brand loyalty by improving user satisfaction and task efficiency. This finding suggests that behavioral-level enhancements exert a positive influence on users’ emotional evaluations and long-term identification at the reflective level. For example, it was demonstrated by Nebel et al. (2017). that behavioral-level design, optimized through increased challenge and the provision of immediate feedback, was found to significantly improve student engagement and learning outcomes, and to foster sustained interest in and positive attitudes toward the learning content. This result has been regarded as empirical evidence supporting the hypothesis that the behavioral level positively influences the reflective level. In summary, it has been consistently demonstrated by existing research that the following hypotheses are supported:

*H1*: The instinctive level (IL) is hypothesized to exert a positive influence on the behavioral level (BL)

*H2*: The behavioral level (BL) is hypothesized to exert a positive influence on the reflective level (RL).

It has been identified by Triberti et al. (2017). that emotional factors have been recognized as essential components in effective design, the elicitation of perceived fun, and the pro-motion of technology use. Specifically, designs with aesthetically pleasing features—such as the phenomenon commonly known as the “wow effect”—have been found to elicit user curiosity and interest (Desmet et al., 2007). Furthermore, gamification mechanisms, including rewards and achievements, together with engaging interactive storylines, have been shown to further enhance users’ perceived enjoyment (Adams et al., 2012). Therefore, visual appeal and intuitive perception are considered to positively influence perceived fun. For instance, educational games and augmented reality (AR) applications (Alexiou et al., 2012). are used to foster emotional engagement by encouraging user participation. Additional approaches have included gamification strategies and the integration of game-based elements into user interfaces (e.g., rewards, achievements), as well as interactive storytelling, in which interactions are embedded within emotionally engaging contexts featuring compelling characters, events, and motivations (Neal, 2012). Accordingly, operational experience and functional satisfaction are regarded as positively contributing to perceived enjoyment. In educational settings, technological self-efficacy (TSE) is defined as an increased sense of competence experienced by students after the successful use of technological tools to complete tasks. This enhancement has been found to enhance perceived trust in such tools (e.g., ICT), which in turn facilitates their acceptance and use (Xu et al., 2024). The influence of per-ceived risk and subjective norms on users’ intention to trust and adopt cloud technologies has also been examined. It has been found that users’ successful task completion using cloud-based systems enhances their sense of achievement, which, in turn, reinforces trust in the technology (Ho et al., 2017). Based on these findings, the following hypotheses have therefore been proposed:

*H3*: The instinctive level (IL) is hypothesized to positively influence perceived fun (PF).

*H4*: The behavioral level (BL) is hypothesized to positively influence perceived enjoyment (PE).

*H5*: The reflective level (RL) is hypothesized to positively influence perceived trust (PT).

#### 2.6.2 Technology acceptance model (TAM)

Based on survey data and statistical analysis conducted among secondary school teachers in Flanders, De Smet et al. (2012) reported that perceived usefulness (PU) had a significant positive impact on learning motivation, learning attitude, and learning satisfaction. These findings suggest that teachers tend to adopt more favorable attitudes toward the use of learning management systems (LMSs) when they perceive them as useful. Perceived usefulness is therefore considered a key factor influencing teachers’ attitudes toward LMS adoption, thereby promoting greater acceptance and intention to use the system. Similarly, other studies have demonstrated that both perceived usefulness (PU) and perceived ease of use (PEOU) are significant predictors of students’ behavioral intentions to use learning systems such as e-portfolios, thus offering empirical support for enhancing user acceptance of educational technology systems (Abdullah et al., 2016). Moreover, perceived usefulness (PU) has been shown to directly influence users’ attitudes toward use and their initial intentions to adopt technological systems (Karahanna and Straub, 1999). Continued usage intentions, within long-term use contexts, are primarily influenced by both PU and user satisfaction, as suggested by the Expectation-Confirmation Model (ECM) (Bhattacherjee, 2001; Praveena and Thomas, 2014). Perceived ease of use (PEOU) is considered a critical factor influencing users’ acceptance of technology. It has been demonstrated in prior research that PEOU is capable of indirectly enhancing perceived usefulness and usage intention by lowering cognitive load and improving system intuitiveness and accessibility (Toros et al., 2024). Moreover, intrinsic motivation (IM) and flow experience are recognized as multiple mediators in the mechanism by which PU, through PEOU, influences learning motivation and learning attitude. These findings further confirm that PU and PEOU are not only associated with more favorable attitudes toward the system but are also shown to indirectly strengthen emotional engagement and usage intention (Wang et al., 2022). Accordingly, the following hypotheses have been formulated:

*H6*: Perceived ease of use (PEOU) is expected to exert a positive influence on perceived usefulness (PU).

*H7*: Perceived usefulness (PU) is expected to positively impact attitude toward use (ATT).

*H8*: Attitude toward use (ATT) is assumed to positively affect interest-driven motivation (IM).

#### 2.6.3 Perceived fun (PF)

Clark and Mayer (2023) demonstrated that learners’ interest, attention, and memory retention are significantly influenced by perceived fun through engaging design elements such as color and anthropomorphic features. The active stimulation of fun has been found to increase student interest by fulfilling users’ core psychological needs, thereby enhancing learning motivation (Pekrun et al., 2007). Lee and Jun (2005) suggested that individuals are intrinsically motivated to engage more with mobile technologies through enjoyable activities. Similarly, fun-oriented design that satisfies users’ fundamental needs has been shown to enhance positive user experiences, evoke intrinsic interest, and foster sustained engagement. These findings provide empirical support for the hypothesis that perceived fun positively influences interest-driven motivation (Mitchell et al., 2020; Liu, 2025). Accordingly, the following hypothesis is proposed:

*H9*: Perceived fun (PF) is hypothesized to positively influence interest-driven motivation (IM).

#### 2.6.4 Perceived enjoyment (PE)

Perceived enjoyment (PE) is recognized as a form of intrinsic motivation (Lee et al., 2005). When a behavior is associated with pleasure and enjoyment, such behavior is more likely to be engaged in by individuals. This implies that the adoption of a technology may be influenced by the enjoyment experienced during its use (Teo et al., 1999). For instance, Davis et al. demonstrated that users’ willingness to use word processing applications was significantly influenced by perceived enjoyment (Triandis, 1979). Similarly, other studies have indicated that interest-driven behaviors are significantly shaped by perceived enjoyment. By fulfilling motivational needs and enhancing output quality, human–computer games (HCGs) have been demonstrated to enhance enjoyment while simultaneously promoting sustained interest and engagement (Pe-Than et al., 2014). Additionally, it has been suggested that students’ interest-driven behaviors can be significantly enhanced through the use of programmable robots, which increase enjoyment during the learning process. This interest has been found to promote deeper engagement and the development of skills such as collaboration and creativity within learning activities (Alvarez and Larrañaga, 2016). Accordingly, the following hypothesis is proposed:

*H10*: Perceived enjoyment (PE) is hypothesized to positively influence interest-driven motivation (IM).

#### 2.6.5 Perceived trust (PT)

Perceived trust (PT) is widely recognized as a key motivational factor underlying interest-driven behavior. When educational tools are perceived as reliable and effective—characterized by collaborative attitudes, a sense of empowerment, and engaging programming interfaces—trust has been found to reduce usage anxiety, increase tool reliance, and foster user interest, thereby promoting learning engagement (Kong et al., 2018). Trust has also been conceptualized as both an affective state and a relational mechanism within social interaction, wherein interest has been shown to be indirectly shaped by trust through emotional responses such as comfort and satisfaction (Lahno, 2001). Research on virtual communities has similarly revealed that mechanisms supporting sustained content contribution—particularly those linked to perceived social status and trust-based incentive structures—are known to influence user motivation for sustained participation. From the perspective of motivational theory, these findings provide additional empirical support for the proposition that perceived trust is positively associated with inter-est-driven behavior (Dong et al., 2020). Accordingly, the following hypothesis is proposed:

*H11*: Perceived trust (PT) is hypothesized to positively influence interest-driven moti-vation (IM).

### 2.7 Conceptual framework

**Figure 1:**
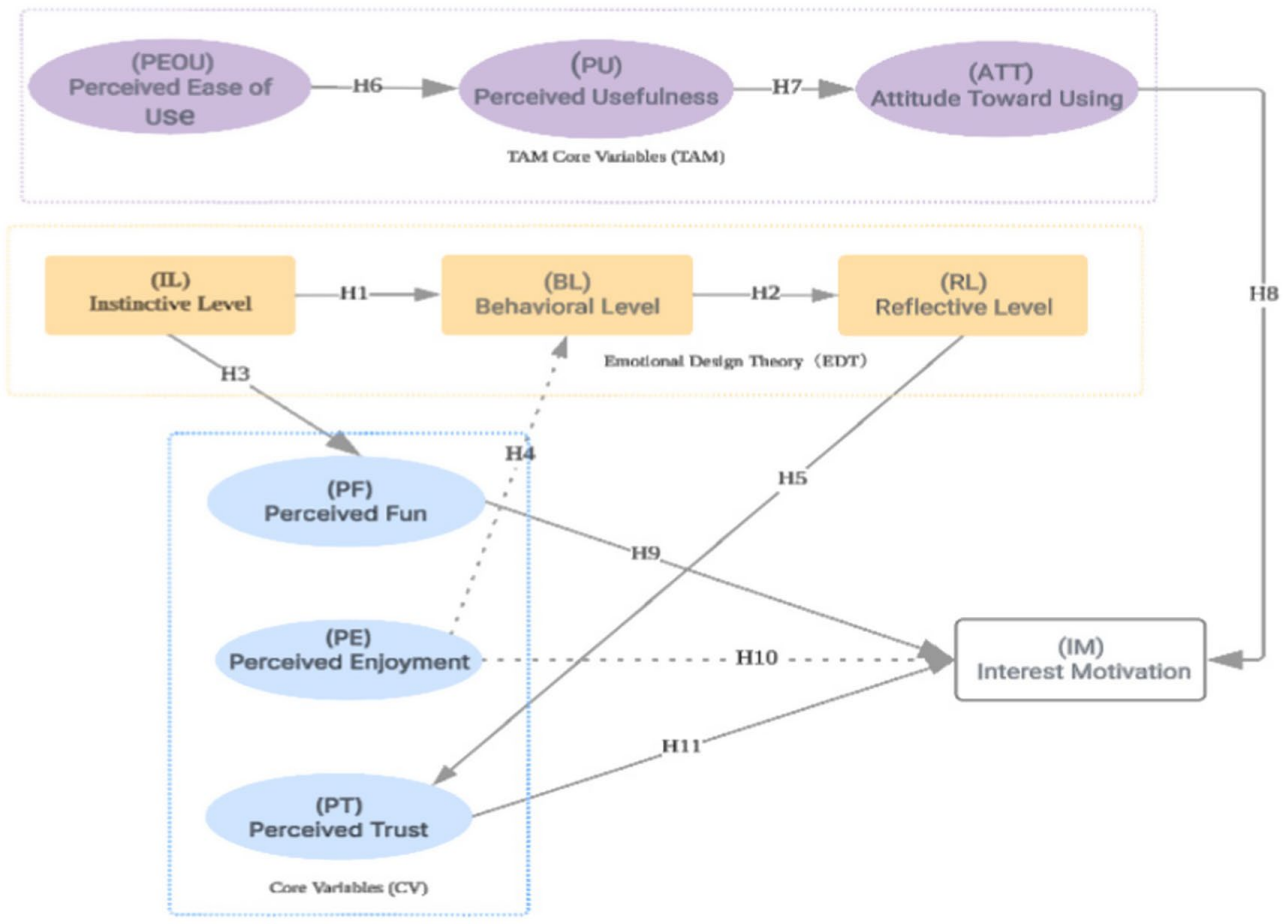
Hypothesized path model.

An interest-driven model was developed based on eleven hypotheses, integrating the Technology Acceptance Model (TAM) with Emotional Design Theory to examine the application of generative AI-based painting in art therapy (Figure 1). The model includes key constructs such as perceived usefulness (PU), perceived ease of use (PEOU), attitude toward use (ATT), behavioral intention (BI), and emotional dimensions including perceived fun, enjoyment, and trust. It systematically illustrates the structural pathways and interrelationships that influence adolescents’ adoption of AI technology in therapeutic settings.

## 3 Method

### 3.1 Research procedures and measurement instruments

The methodology of this study was divided into six key phases to ensure methodological coherence from tool selection to model validation: (1) An integrated analytical framework combining emotional design theory and the Technology Acceptance Model (TAM) was formulated and examined. A model of interest-driven factors influencing adolescents’ engagement was constructed, accompanied by eleven hypothesized structural paths. (2) The experimental tool and theme were established by identifying ChatGPT-DALL·E as the AI painting platform and selecting “My Childhood” as a unified creative theme. The emotional resonance of the theme was validated via expert consultation and a comprehensive literature review (Wildschut et al., 2006). (3) Sample recruitment and questionnaire development involved the random selection of 444 adolescents from a secondary school in Hubei Province, who were assigned to either a traditional painting group or an AI-assisted painting group. A 35-item Likert-scale questionnaire was designed to capture key variables and was refined through back-translation, expert review, and pilot testing to ensure psychometric soundness. (4) Structural equation modeling (SEM) was applied to analyze the survey data. SPSS and AMOS were used to evaluate the measurement model and assess the significance of hypothesized path relationships, along with the reliability and validity of the overall structural model. (5) Variables supported by SEM results were subsequently fed into an artificial neural network (ANN) model to assess the robustness of the findings under nonlinear conditions and to calculate the relative importance of each independent variable in predicting the outcome variable. (6) Finally, results from both SEM and ANN analyses were integrated to explore the interest-driven mechanisms of generative AI painting in adolescent art therapy, and practical recommendations were offered for future tool development, educational practice, and psychological intervention.

#### 3.1.1 Measurement instruments

**Figure 2:**
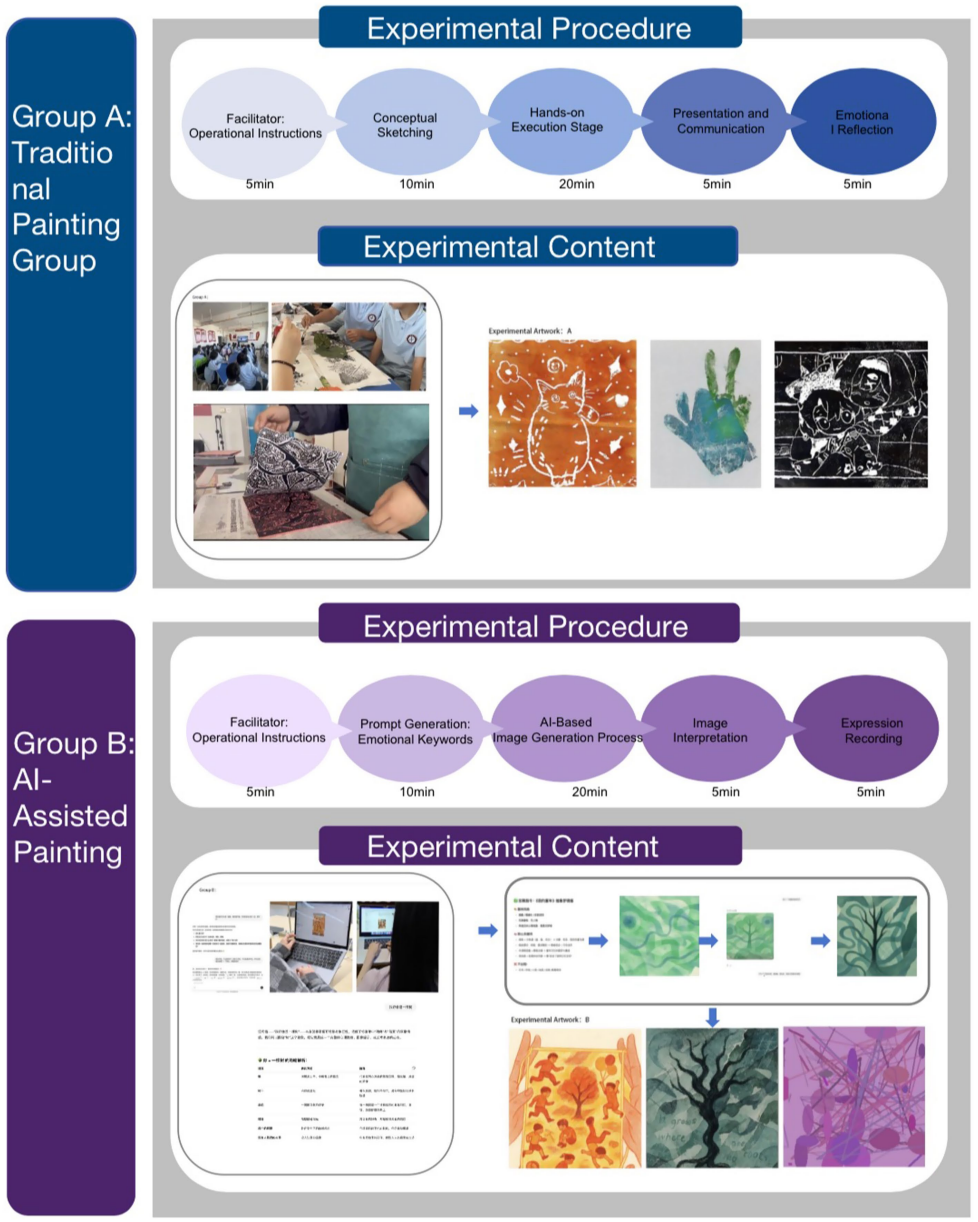
Experimental process and representative artworks.

In the experimental design, multiple dimensions were taken into account to ensure the methodological rigor and practical applicability of the research instruments, experimental theme, and measurement methods (Figure 2). Specifically, ChatGPT-DALL·E was chosen as the AI painting platform due to three key advantages. First, the platform is characterized by a high degree of interactivity and immediate visual feedback, enabling adolescents to generate visual outputs in real time during the creative process, which enhances creative engagement and emotional expression. Second, a simplified interface and intuitive functionality make it possible to automatically generate personalized artwork based on user-generated textual prompts, thereby greatly reducing the barrier to entry and supporting participation even among adolescents with no prior artistic experience. Finally, given adolescents’ generally high levels of digital literacy and technological acceptance, the use of DALL·E is perceived as engaging and immersive, thereby contributing to sustained interest and the development of intrinsic motivation (Ramesh et al., 2022).

### 3.2 Development of the questionnaire and recruitment of participants

A structured cross-sectional survey design was adopted to systematically investigate the interest-driven factors influencing adolescents’ engagement with generative artificial intelligence (AI) painting tools within the context of art-based therapy (Fowler, 2013). The target population comprised school-enrolled adolescents aged 10 to 19 in Hubei Province, China. Participants were recruited from multiple middle and high schools using a random sampling method (Nanney et al., 2016). Adolescents were selected as the focal demographic due to their developmental sensitivity to cognitive and emotional stimuli, as well as their high receptivity to digital technologies.

The survey was administered via an online questionnaire platform. While the digital format facilitated centralized data collection and efficient management, informed consent was obtained from each participant prior to participation. To ensure sample representativeness and enhance data reliability, multiple quality control procedures were implemented: (1) A screening item at the beginning of the survey was used to exclude individuals who had never engaged in any form of art therapy. (2) A reverse-coded attention-check question was included at the end of the survey; failure to identify this item resulted in exclusion from analysis. (3) Responses exhibiting uniform answers across all items were removed. (4) Surveys completed in under 60 s were deemed inattentive and were excluded from the dataset. These procedures were designed to mitigate common self-reporting biases and reduce the effects of social desirability.

The measurement instruments employed in this study were adapted from constructs extensively validated in prior literature (see Table 1), encompassing 11 latent variables grounded in the Technology Acceptance Model (TAM) and Emotional Design Theory (EDT), with a total of 33 measurement items. The questionnaire was originally developed in English and subsequently subjected to a bilingual translation and back-translation process to ensure semantic consistency and cultural relevance. Two rounds of pilot testing were conducted with adolescent participants (n₁ = 23; n₂ = 36) prior to the formal administration of the survey. Following item refinement, the internal consistency of the instrument, as indicated by Cronbach’s alpha, improved from an initial value of 0.743 to 0.812, confirming a satisfactory level of reliability. The finalized questionnaire consisted of two sections: the first gathered demographic data, while the second employed a five-point Likert scale to assess participants’ attitudes, trust, behavioral intentions, and emotional responses related to AI painting tools. All surveys were conducted in classroom settings under the supervision of trained research personnel, with each session lasting approximately 45 min.

**Table 1:** Measurement variables and their references.

Dimension	Code and content	Sources
Instinctive level (IL)	IL1: The overall user interface design of the selected tool is satisfactory. IL2: The layout of the selected tool is well-structured and easy to use. IL3: IL3: I instinctively felt that this tool suited me well and was eager to try it with little hesitation.	Norman (2007), Cho et al. (2009), Azuaje et al. (2023)
Behavioral level (BL)	BL1: Most tasks could be completed smoothly while using the tool, with few obstacles encountered. BL2: The interaction process with the AI art tool was timely and informative, which motivated me to continue using it. BL3: I actively explored additional functions of the tool to better support artistic creation or emotional adjustment.	Norman (2007), Azuaje et al. (2023)
Reflective level (RL)	RL1: After using the tool for some time, I reflected on the psychological or creative gains it provided. RL2: I contemplated the role this tool played in my emotional expression or artistic style development. RL3: On a deeper level, I acknowledged its significance for my personal growth or aesthetic appreciation.	Norman (2007), Azuaje et al. (2023)
Perceived ease of use (PEOU)	PEOU1: The interface provides easily understandable information. PEOU2: The tool is very simple to use. PEOU3: I encountered no difficulty in using this tool.	Norman (2007), Azuaje et al. (2023), Fan and Jiang (2024)
Perceived usefulness (PU)	PU1: I consider the Generative AI Painting tool to be useful. PU2: The use of this technology improves efficiency. PU3: With the help of this tool, the quality of my creative environment has been enhanced.	Azuaje et al. (2023), Davis (1989), Fan and Jiang (2024), Abdullah et al. (2016)
Attitude toward use (ATT)	ATT1: My overall experience with this Generative AI Painting tool has been positive and enjoyable. ATT2: Under similar conditions, I would prefer to continue using this tool for Digital Art Therapy or emotional management. ATT3: After the first use, I intend to continue using the application.	Davis (1989), Azuaje et al. (2023), Fan and Jiang (2024), Abdullah et al. (2016)
Perceived fun (PF)	PF1: The tool brought novel experiences that encouraged me to explore various creative approaches. PF2: I became fully engaged during the use of the tool. PT3: I am confident in the tool’s long-term availability, continuous optimization, and sustained effectiveness.	Fan and Jiang (2024), Abdullah et al. (2016)
Perceived enjoyment (PE)	PE1: I experienced immersive enjoyment and satisfaction when co-creating art with AI. PE2: Using the AI-assisted drawing tool made the design process easier and more pleasant, reducing creative stress. PE3: I deeply enjoyed the process of using AI drawing tools for Digital Art Therapy, which provided a sense of fulfillment and achievement.	Abdullah et al. (2016), Gao et al. (2023)
Perceived trust (PT)	PT1: I trust that the tool securely protects my personal information and artwork.	Orr (2015), Wang and Chen (2024)
	PT2: The tool was perceived as stable and reliable, with few errors or crashes	
	PT3: I am confident in the tool’s long-term availability, continuous optimization, and sustained effectiveness.	
Interest motivation (IM)	IM1: The novelty and wonder I experienced while using the AI tool inspired me to explore more functions and modes of artistic expression.	Kaimal et al. (2020), Webster et al. (1993)
	IM2: I am willing to invest more time and energy in using this AI tool, as it sparked my interest and offered emotional release.	
	IM3: After experiencing positive emotions from using the AI tool, I am more inclined to use it again in the future to maintain or enhance these feelings.	

#### 3.2.1 Recruitment of participants

Following a rigorous quality control process, a total of 508 questionnaires were collected, among which 444 were determined to be valid, resulting in an effective response rate of 87.35%. This sample size surpassed the commonly accepted threshold for Structural Equation Modeling (SEM), which recommends a minimum of ten respondents per observed variable, thereby ensuring adequate statistical power for model estimation. The demographic profile of the participants is presented in Table 2. Among the respondents, 46.76% were male (*n* = 208) and 53.24% were female (*n* = 236). In terms of developmental stages, 58.75% were aged 15–17 (mid-adolescence), 15.82% were aged 10–14 (early adolescence), and 25.43% were aged 18–19 (late adolescence). Regarding educational level, 15.43% were primary school students, 47.23% were enrolled in junior high school, and 37.34% were attending senior high school.

**Table 2:** Demographic characteristics of respondents.

Attribute	Items	Numbers	Percentage (%)
Gender	Male	208	46.76**%**
	Female	236	52.24**%**
Adolescent developmental stage	Early Adolescence (10–14 years)	70	15.82**%**
	Middle Adolescence (15–17 years)	261	58.75**%**
	Middle Adolescence (15–17 years)	113	25.43**%**
Educational level	Primary School	69	15.43**%**
	Junior High School	210	47.23**%**
	Senior High School	165	37.34**%**
Experience with AI tools	Yes	250	56.37**%**
	No	194	43.63**%**
Experience with digital art therapy	Yes	283	63.75**%**
	No	161	36.25**%**

In addition, 56.37% of participants reported previous experience with AI tools, and 63.75% indicated some degree of exposure to art therapy. These findings suggest that the sample possessed substantial psychological and technological readiness, making it highly suitable for the objectives of this study. For missing values (less than 5% of total responses), mean substitution was employed to maintain data completeness. Outliers were retained due to the use of Artificial Neural Network (ANN) modeling in subsequent analyses, as ANN is recognized for its robustness to extreme values. The inclusion of outliers was expected to improve the model’s generalizability and predictive performance.

All procedures adhered strictly to ethical research standards. Prior to participation, all individuals were fully informed of the study’s purpose, their rights, and the voluntary nature of their involvement, including the right to withdraw at any point. Anonymity was maintained throughout the data collection and analysis phases to ensure maximum protection of participants’ privacy and confidentiality.

### 3.3 Data analysis and empirical findings

#### 3.3.1 Evaluation of the measurement model

SPSS 26.0 and AMOS 28.0 were employed as the primary analytical tools in this study. During the reliability testing phase, each construct of the questionnaire underwent internal consistency and composite reliability assessments. According to the criteria proposed by Chin (1998), a scale can be regarded as demonstrating acceptable convergent validity if its factor loadings and composite reliability (CR) exceed 0.70, and the average variance extracted (AVE) is greater than 0.50. Based on these standards, as shown in Table 3, all AVE values for the measured constructs were above 0.50, and all CR values exceeded 0.70 (e.g., AVE = 0.868 and CR = 0.950 for perceived trust; AVE = 0.864 and CR = 0.948 for interest-driven behavior), indicating that the items effectively explained their respective latent constructs and confirming internal consistency. All factor loadings were statistically significant (*p* < 0.001), with critical ratios greater than 1.96. Specifically, the factor loadings for the visceral level ranged from 0.799 to 0.843, and for perceived enjoyment, from 0.687 to 0.872, indicating strong factor validity. Furthermore, Cronbach’s alpha coefficients for all constructs exceeded the recommended threshold of 0.70 (ranging from 0.765 to 0.878), further supporting internal consistency.

**Table 3:** Results of confirmatory factor analysis: AVE and CR values.

Variables	Code	Mean	S.D.	Estimate	C.R	P	α	AVE	CR
PEOU	PEOU1	3.392	1.383	0.572					
	PEOU2			0.553	8.285	***	0.895	0.581	0.777
	PEOU3			0.615	8.721	***			
PU	PU1	3.299	1.279	0.542					
	PU2			0.561	8.450	***	0.941	0.540	0.743
	PU3			0.515	8.023	***			
ATT	ATT1	3.327	1.318	0.615					
	ATT2			0.631	10.524	***	0.937	0.616	0.804
	ATT3			0.601	10.181	***			
IL	IL1	3.237	1.373	0.799					
	IL2			0.807	18.148	***	0.953	0.817	0.926
	IL3			0.843	19.010	***			
BL	BL1	3.289	1.327	0.655					
	BL2			0.617	10.862	***	0.921	0.638	0.820
	BL3			0.641	11.167	***			
RL	RL1	3.407	1.274	0.636					
	RL2			0.554	9.761	***	0.899	0.595	0.788
	RL3			0.593	10.269	***			
PF	PF1	3.476	1.257	0.687					
	PF2			0.869	17.805	***	0.854	0.814	0.924
	PF3			0.872	17.870	***			
PE	PE1	2.674	1.281	0.851					
	PE2			0.748	15.388	***	0.917	0.779	0.907
	PE3			0.733	15.214	***			
PT	PT1	3.395	1.315	0.863					
	PT2			0.875	26.272	***	0.922	0.868	0.950
	PT3			0.866	25.804	***			
IM	IM1	3.749	1.192	0.814					
	IM2			0.854	22.530	***	0.935	0.864	0.948
	IM3			0.921	24.773	***			

During the validity assessment phase, three types of validity were evaluated: content validity, convergent validity, and discriminant validity (Straub et al., 2004). Discriminant validity was confirmed by the observation that, for each construct, the square root of its AVE was greater than the Pearson correlation coefficients with other constructs, as shown in the corresponding rows and columns in Table 4.

**Table 4:** Results of discriminate validity of the research model.

Variables	PEOU	PU	ATT	IL	BL	RL	PF	PE	PT	IM
PEOU	0.762									
PU	0.398**	0.735								
ATT	0.478**	0.430**	0.785							
IL	0.352**	0.404**	0.375**	0.904						
BL	0.358**	0.458**	0.390**	0.692**	0.799					
RL	0.527**	0.545**	0.705**	0.396**	0.450**	0.772				
PF	0.408**	0.450**	0.476**	0.471**	0.501**	0.503**	0.902			
PE	−0.067	0.049	−0.040	0.041	0.039	−0.048	0.010	0.883		
PT	0.415**	0.422**	0.500**	0.438**	0.488**	0.505**	0.819**	−0.021	0.932	
IM	0.471**	0.488**	0.511**	0.543**	0.567**	0.547**	0.593**	0.021	0.591**	0.930

### 3.4 Model fit and hypothesis testing

In accordance with the recommendations of Hair et al. (2012), the chi-square to degrees of freedom ratio (χ^2^/df) was considered acceptable when maintained below a value of 5. Additionally, model fit was evaluated based on the criteria proposed by Hu and Bentler (1999). The model was estimated using the maximum likelihood method and yielded fit indices meeting the established thresholds: the chi-square value was 470.945, with a χ^2^/df ratio of 1.308, both within the recommended range. The RMSEA was 0.025, which fell well below the threshold typically applied in confirmatory factor analysis. Furthermore, the AGFI was 0.942, the TLI was 0.979, and the CFI was 0.984, all exceeding the commonly accepted threshold of 0.80. As indicated in Table 5, the structural model exhibited a satisfactory overall fit to the data.

**Table 5:** Model fit indices.

Model	X^2^	X^2^/DF	AGFI	TLI	CFI	RMSEA
Confirmatory factor model	470.945	1.308	0.942	0.979	0.984	0.025
Structural equation model	1214.248	3.090	0.879	0.857	0.879	0.064
Recommended threshold	*p* > 0.05	1 ~ 5	>0.8	>0.8	>0.8	<0.08

**Figure 3:**
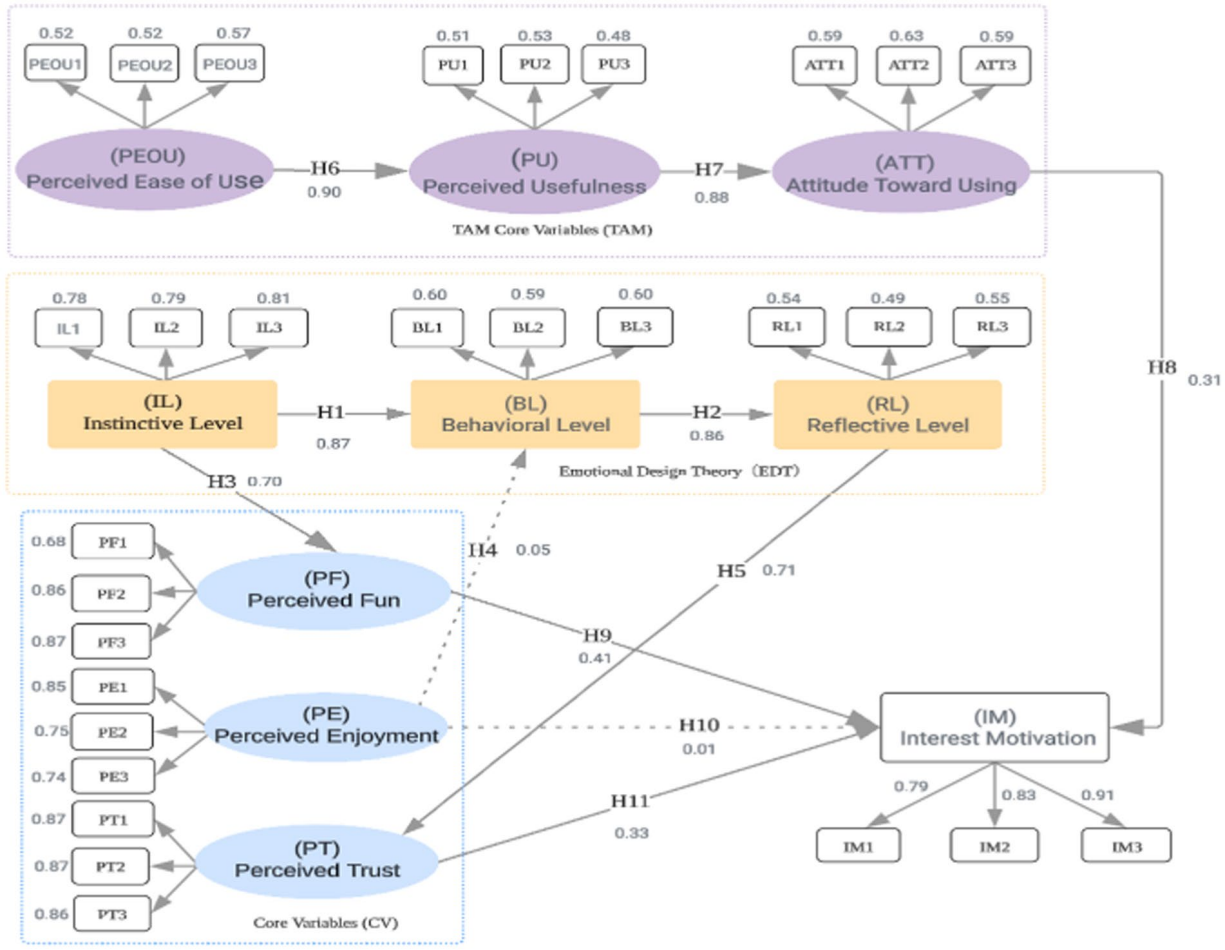
Model path analysis results.

As shown in Table 6 and Figure 3, the path coefficients for H1, H2, H3 (*p* = 0.023), H5, H6, H7, H9, and H11 (*p* = 0.043) were found to be statistically significant. These results suggest that H1 (the effect of the visceral level on the behavioral level), H2 (the effect of the behavioral level on the reflective level), H3 (the effect of the visceral level on perceived enjoyment), H5 (the effect of the reflective level on perceived trust), H6 (the effect of perceived ease of use on perceived usefulness), H7 (the effect of perceived usefulness on attitude toward use), H9 (the effect of perceived enjoyment on interest-driven behavior), and H11 (the effect of perceived trust on interest-driven behavior) all exerted significant positive effects.

**Table 6:** Structural equation modeling (SEM) path analysis results.

Hypothesis	Path	Estimate	S. E.	C. R.	P	Result
H1	IL → BL	0.868	0.056	11.630	***	Supported
H2	BL → RL	0.860	0.091	8.866	***	Supported
H3	IL → PF	0.703	0.056	13.639	0.023	Supported
H4	BL → PE	−0.054	0.065	−1.001	0.317	Rejected
H5	RL → PT	0.711	0.110	9.557	***	Supported
H6	PEOU → PU	0.896	0.113	7.290	***	Supported
H7	PU → ATT	0.876	0.130	7.959	***	Supported
H8	ATT → IM	0.310	0.064	6.320	0.037	Supported
H9	PF → IM	0.406	0.040	9.348	0.015	Supported
H10	PE → IM	−0.013	0.041	0.367	0.713	Rejected
H11	PT → IM	0.331	0.039	8.162	0.043	Supported

In contrast, the path coefficients for H4 (*p* = 0.037) and H10 were not statistically significant, indicating that the hypothesized effects of the behavioral level on perceived enjoyment, attitude toward use on interest-driven behavior, and perceived enjoyment on interest-driven behavior were not empirically supported.

### 3.5 Artificial neural network analysis

To enhance the robustness of the Structural Equation Modeling (SEM) results and to identify potential nonlinear relationships within the mechanism underlying adolescent interest-driven engagement, an Artificial Neural Network (ANN) analysis was employed. The incorporation of ANN was primarily motivated by its ability to model complex, nonlinear interactions among variables, thereby addressing the inherent limitations of SEM, which relies on linear assumptions. Recent studies in domains such as metaverse-based education and generative AI-supported learning (Ali et al., 2025; Soliman et al., 2024) have demonstrated that dual-stage SEM–ANN models offer superior explanatory power and generalizability in predicting behavior and modeling perceptual mechanisms.

A feedforward Multilayer Perceptron (MLP) architecture was adopted, comprising an input layer, a hidden layer with 10 neurons, and an output layer. The sigmoid activation function was applied, and the backpropagation algorithm was used for optimization. The learning rate was set to 0.01, and the training was conducted over 500 epochs. All input variables were normalized to a 0–1 range prior to training to enhance convergence speed and model stability. To prevent overfitting and improve predictive accuracy, 10-fold cross-validation was used during the training and evaluation phases.

The input variables consisted of the four significant predictors identified in the SEM analysis: Perceived Fun (PF), Perceived Enjoyment (PE), Perceived Trust (PT), and Attitude Toward Use (ATT). The output variable was Interest-Driven Motivation (IM). The dataset was randomly partitioned into a training set (90%, *n* = 399) and a testing set (10%, *n* = 45). Predictive performance was assessed using the Root Mean Square Error (RMSE). As reported in Table 7, the average RMSE was 0.261 for the training set and 0.292 for the testing set, indicating stable and consistent predictive capability across data subsets.

**Table 7:** RMSE value of artificial neural network.

ANN	Model A	
	Input: PF, FE, PT, ATT	
	Ouput: IM	
	Training (90)	Testing (10)
ANN1	0.244	0.353
ANN2	0.260	0.146
ANN3	0.277	0.159
ANN4	0.284	0.504
ANN5	0.258	0.251
ANN6	0.272	0.178
ANN7	0.303	0.327
ANN8	0.236	0.234
ANN9	0.249	0.379
ANN10	0.235	0.392
Mean	0.261	0.292
SD	0.022	0.117

To further assess the contribution of each input variable to the output prediction, a sensitivity analysis was conducted on the trained ANN model. As shown in Table 8, Attitude Toward Use (ATT) emerged as the most influential predictor (normalized importance = 100%), followed by Perceived Fun (PF, 97.9%) and Perceived Trust (PT, 93.4%). In contrast, Perceived Enjoyment (PE) demonstrated the lowest importance (19.3%), suggesting that despite its theoretical relevance, its actual impact on interest activation in this context was limited.

**Table 8:** Neural network sensitivity analysis.

Model A (Output: IM)				
Neural network	PF	FE	PT	ATT
ANN1	0.300	0.090	0.281	0.329
ANN2	0.361	0.063	0.228	0.348
ANN3	0.294	0.065	0.357	0.284
ANN4	0.263	0.081	0.346	0.310
ANN5	0.252	0.068	0.379	0.301
ANN6	0.276	0.042	0.342	0.340
ANN7	0.375	0.044	0.151	0.430
ANN8	0.335	0.048	0.368	0.248
ANN9	0.361	0.077	0.200	0.363
ANN10	0.336	0.042	0.354	0.267
Average importance	0.315	0.062	0.306	0.321
Normalized importance (%)	97.919	19.254	93.354	100.000

Additionally, as illustrated in Table 9, the relative importance rankings of predictors were largely consistent between the SEM and ANN models. While Perceived Fun (PF) was identified as the most significant predictor in the SEM model, Attitude Toward Use (ATT) ranked highest in the ANN model. This variation underscores the methodological complementarity of the two approaches: SEM is more appropriate for hypothesis testing and theoretical validation, whereas ANN is better suited for uncovering nonlinear predictive strengths in complex behavioral data.

**Table 9:** Comparison of SEM and ANN results.

Model 1 (Output: BI)	SEM: path coefficient	SEM ranking	ANN: normalized importance (%)	ANN ranking	Consistency analysis
PF	0.410	1	97.919	2	Highly consistent
PE	0.010	4	19.254	4	Perfectly
PT	0.330	2	93.354	3	Perfectly
ATT	0.310	3	100.000	1	Highly consistent

## 4 Discussion

### 4.1 Overview of key findings

An empirical investigation was conducted into the interest-driven mechanism of generative AI painting within the context of adolescent art therapy. The study was centered on the predictive effects of TAM model pathways, the three-level emotional design framework, and affective cognitive factors in predicting user interest. A dual-model cross-validation approach was adopted, involving both structural equation modeling (SEM) and artificial neural networks (ANN). Overall, the analytical results showed statistical support for most of the hypothesized paths, demonstrating adolescents’ multidimensional responses to tool design, perceptual experience, and psychological trust during the digital art creation process.

With respect to Research Question 1—"According to the TAM model, to what extent is adolescents’ interest shaped by perceived ease of use, perceived usefulness, and attitude toward use?”—the current study demonstrated a significant effect of perceived ease of use (PEOU) on perceived usefulness (PU) (H6) and a strong positive effect of PU on attitude toward use (ATT) (H7). Moreover, ATT was shown to significantly influence interest-driven motivation (H8). These findings are consistent with pathway models identified in TAM-related studies by Abdullah et al. (2016), Davis (1989), and Ho et al. (2017), indicating that when adolescents perceive AI painting tools as easy to use and useful, they are more likely to remain engaged. The ANN model further revealed the primacy of attitude toward use in predicting interest, with ATT showing the highest normalized importance (100%), thereby reinforcing the core role of the “attitude–behavioral intention” pathway within the TAM framework.

Second, in relation to Research Question 2— “Within the three-level framework of emotional design theory, how is emotional engagement stimulated by AI painting tools among adolescents?”—a significant influence was observed from the visceral level (IL) to the behavioral level (BL) (H1), followed by a subsequent influence of the behavioral level on the reflective level (RL) (H2). These three levels were found to indirectly affect interest-driven motivation through perceived fun (PF), perceived enjoyment (PE), and perceived trust (PT), respectively. In particular, the paths involving PF and PT (H9 and H11) were strongly supported empirically and were ranked among the top paths in both SEM and ANN analyses. These findings provide empirical support for the affective progression mechanism proposed by Norman (2007), wherein emotional engagement evolves from intuitive perception to long-term identification. The results are further aligned with Chang and Chen (2024), empirical validation of the visceral–behavioral linkage, as well as Hassenzahl and Tractinsky (2006)‘s model of positive affect transformation between behavioral and reflective levels. These results. suggest that immediate visual appeal and interaction fluency serve as foundational elements in adolescents’ digital creation experiences, while internal meaning and self-reflection constitute critical drivers of long-term engagement.

In response to Research Question 3—“How do perceived fun, enjoyment, and trust contribute to the attractiveness of AI painting tools for adolescents?”—significant associations were identified in the SEM analysis between perceived fun (PF), perceived trust (PT), and interest-driven motivation (H9, H11), and were further supported by ANN results, where normalized importance values for both variables exceeded 90%. These findings suggest that PF and PT serve as stable and influential predictors in adolescents’ interest formation mechanisms. The results are consistent with the conclusions of Abdullah et al. (2016) and Lee and Jun (2005), who highlighted the importance of enjoyment in increasing usage intention and also support the theoretical proposition by Bhattacherjee (2001) regarding the positive relationship between tool trust and educational acceptance.

### 4.2 Interpretation of pathway results

Although most of the hypothesized paths in this study were statistically supported, several failed to reach significance and therefore warrant further clarification to improve the clarity of the findings and strengthen their theoretical implications.

Hypothesis H4 (Behavioral Layer → Perceived Enjoyment) was not supported in the structural equation model, indicating that the behavioral design characteristics of generative AI painting tools—including system fluency and operational feedback—had limited influence on adolescents’ perceived enjoyment. While this finding diverges from earlier research on traditional interactive platforms (e.g., Lee et al., 2005), it aligns with more recent investigations into user experiences with generative AI systems. According to Soliman et al. (2024), in highly automated generative AI environments, users’ perceived sense of control is substantially diminished, and enjoyment tends to depend more on pre-input visual stimuli or post-output interpretive engagement than on process-oriented interactivity.

Hypothesis H10 (Perceived Enjoyment → Interest-Driven Motivation) was also not supported in the structural equation model. Although Perceived Enjoyment (PE) has been extensively recognized as a key predictor of behavioral intention in the technology acceptance literature (e.g., Davis, 1989; Venkatesh and Bala, 2008), no statistically significant path from PE to Interest-Driven Motivation (IM) was observed in this study. This outcome suggests that the effect of PE on adolescents’ interest-driven engagement in generative AI–assisted art creation may be subject to specific contextual or boundary conditions.

Overall, the core findings of this study are corroborated by a growing body of recent literature. For instance, Soliman et al. (2025), using a two-stage path analysis, identified structural differences in AI usage motivation across age groups. The present study further demonstrates that adolescent users exhibit greater sensitivity to constructs such as Perceived Trust and Perceived Fun than to Ease of Use, suggesting that traditional TAM pathways may require revision through the integration of emotional design principles to ensure their applicability to younger populations. Furthermore, the empirical validation of Norman’s three-level emotional design framework in this study aligns with the immersive emotional interaction mechanisms proposed by Triberti et al. (2017), highlighting a cognitive–affective linkage between the visceral and reflective layers.

Notably, although Perceived Enjoyment (PE) has long been regarded as a central determinant of behavioral intention (e.g., Davis, 1989; Fowler 2013), no statistically significant path from PE to IM was identified in the structural equation model (SEM). Additionally, its normalized importance in the artificial neural network (ANN) analysis was only 19.25%, which was markedly lower than that of Perceived Fun (PF) and Perceived Trust (PT). These results can be interpreted through the following three dimensions:

1. Theoretical Dimension: PE is generally elicited by behavioral-level factors such as interaction fluency and system responsiveness. However, recent studies (e.g., Triberti et al., 2017) suggest that enjoyable experiences are increasingly shaped by sensory stimuli, emotional arousal, and narrative immersion. Elements such as the “wow effect” (Desmet et al., 2007), gamification strategies (e.g., reward feedback) (Adams et al., 2012), and emotionally embedded content (Neal, 2012) have been shown to be particularly effective in fostering enjoyment among adolescents.
2. Technological Dimension: Leading generative AI tools, including ChatGPT and DALL·E, are driven by prompt-based inputs and automated outputs, which limit user interactivity and procedural control. Compared to conventional digital drawing platforms that enable continuous input and real-time feedback, these systems reduce active participation in the creative process, thereby weakening enjoyment derived from behavioral engagement.
3. Demographic Dimension: The sample primarily consisted of adolescents aged 10–19, a group previously found to be highly responsive to novelty, aesthetic appeal, and emotional resonance (e.g., in augmented reality education). Accordingly, their enjoyment is more likely to be driven by visceral-level elements (e.g., visual attraction) and reflective-level engagement (e.g., meaning-making), rather than behavioral-level attributes such as task efficiency or system usability.

### 4.3 Design implications for generative AI tools

In light of these findings, it is imperative that the design of generative AI tools for adolescent users be re-evaluated in terms of how Perceived Enjoyment can be effectively stimulated and translated into sustained usage intentions and creative motivation. Three strategic directions are proposed:

1. Integration of Gamification Mechanisms: Gamified components—such as visual achievement systems, challenge-based tasks, and point-reward structures—should be incorporated to stimulate curiosity and foster a sense of accomplishment among adolescent users. Examples include style unlocking, daily creation challenges, and AI-assisted collaborative drawing, which may be embedded into the creative workflow to enhance engagement, goal orientation, and experiential enjoyment.
2. Enhancement of Dynamic Visual Feedback and Immersive Interactions: Instead of relying solely on static image output, dynamic features such as micro-animated feedback, progressive image generation, and evolutionary playback of the creative process should be implemented to provide continuous visual stimulation. These approaches may compensate for the absence of procedural engagement at the behavioral level while enhancing visceral-level multisensory satisfaction.
3. Developmentally Adaptive and Emotionally Resonant Design Strategies: Considering the cognitive, aesthetic, and emotional variability among adolescents, design strategies should accommodate diverse templates and personalized prompts—including cartoon styles, youth-centric themes, and emotion-driven visual journaling—to foster affective resonance and opportunities for self-expression. These approaches can facilitate meaning construction at the reflective level.

In conclusion, sole reliance on system-based procedural enjoyment is no longer adequate to sustain engagement in generative AI environments. Future design paradigms should shift from operation-centered models toward experience-centered and growth-oriented approaches, reconstructing the logic and context of enjoyment generation to promote long-term motivation and psychological support among adolescent users.

### 4.4 Therapeutic limitations of generative AI

Despite the promising findings, a fundamental limitation should be acknowledged concerning the therapeutic validity of AI-generated art. Traditional art therapy places strong emphasis not only on the final product but, more importantly, on the creative process—characterized by immersive engagement, self-reflection, and the expression of unconscious imagery. However, the automated and prompt-driven nature of generative AI tools, such as DALL·E, may curtail opportunities for self-directed exploration and hinder deep psychological involvement. The partial delegation of creative agency to the algorithm reduces adolescents’ participation in active meaning-making and reflective construction. As a result, the diminished procedural control and constrained expressive autonomy may undermine the depth of therapeutic engagement. To address this limitation, future systems should adopt hybrid approaches that integrate manual input alongside AI-driven features, thereby restoring the balance between algorithmic support and human-centered creativity within the art therapy context.

### 4.5 Ethical considerations

Ethical considerations represent another crucial dimension, particularly given the sensitive nature of psychological intervention contexts. First, privacy concerns must be addressed, especially regarding the handling of user data and generated imagery. Adolescents’ artworks may carry emotional or psychological implications, and clear data protection measures must be implemented. Second, the issue of copyright remains ambiguous in AI-generated content, which could lead to confusion over ownership, authorship, and emotional attachment to the creative product. Lastly, participant autonomy should be safeguarded to ensure that the use of AI tools does not diminish users’ creative control or agency. Explicit consent, transparent data policies, and the optionality of AI assistance are recommended as safeguards to support ethical practice in therapeutic settings.

It is also worth noting that participants in this study had varying levels of prior experience with generative AI tools. While such differences were recorded during demographic profiling, they were not explicitly controlled in the main analysis. Participants unfamiliar with AI systems may have responded more strongly to the novelty and visual immediacy of the experience, potentially inflating scores for perceived fun or trust. Conversely, more experienced users may have developed critical perspectives or expectations based on prior encounters. This variability introduces an interpretive layer that may influence the generalizability of certain findings. Future research could explore subgroup comparisons based on experience level or apply multigroup SEM to assess differential pathway strengths across user profiles.

In summary, this study demonstrated the alignment of core pathways identified in the Technology Acceptance Model (TAM) and emotional design theory, while also highlighting the critical roles of perceived fun and perceived trust in the context of AI-based art therapy. Furthermore, the limitations of certain traditional pathways—such as perceived enjoyment—were clarified, thereby offering both theoretical insights and practical guidance for optimizing emotion-driven mechanisms in the future design of generative AI systems.

## 5 Conclusion

### 5.1 Theoretical implications

Grounded in the integration of emotional design theory (EDT) and the Technology Acceptance Model (TAM), a model of interest-driven factors was developed, and a generative AI painting tool (ChatGPT-DALL·E) was employed as the experimental platform. The study examined the interactions between visceral perception, behavioral experience, reflective cognition, and technology acceptance within the context of digital art therapy. Supported by both structural equation modeling (SEM) and artificial neural network (ANN) analyses, attitude toward use, perceived fun, and perceived trust were identified as the three primary factors driving adolescents’ sustained interest. In contrast, perceived enjoyment—although associated with intrinsic motivation—exhibited relatively weak empirical effects, warranting further investigation. A theoretical gap at the intersection of generative AI and emotional design within adolescent mental health interventions has been addressed. By integrating affective cognition, user attitudes toward technology, and emotion-driven behavior into a unified explanatory model, this study extends the theoretical boundaries of digital therapeutic research. Compared with traditional art therapy approaches, AI-based tools were shown to offer greater interactivity and personalized feedback, while also fostering positive psychological outcomes by enhancing exploratory intention and lowering expressive barriers.

### 5.2 Practical implications

Based on empirical findings from both structural equation modeling (SEM) and artificial neural network (ANN) analyses, actionable guidance has been provided for applying generative AI painting tools in adolescent mental health interventions and educational practices.

First, within school-based educational settings, perceived fun (PF) was identified as a significant factor influencing adolescents’ attitudes toward use (with an ANN importance of 93.4%). Therefore, it is advised that AI painting activities be incorporated into art curricula or emotional education modules, particularly for students with limited verbal expression or social anxiety. For instance, assigning themed tasks such as “AI Emotion Painting” and encouraging students to generate visual imagery using prompts related to their moods may enhance creative motivation and self-expression.

Second, in therapeutic contexts, perceived trust (PT) was found to play a critical role in interest-driven engagement (with an ANN importance of 91.6%). This suggests that therapists should clearly communicate data security protocols and privacy protections when introducing AI tools. It is recommended that a co-creation model be adopted, wherein therapists guide students within a controlled environment. By explicitly stating that personal data will not be stored or analyzed, users’ distrust toward the technology may be alleviated.

Third, attitude toward use (ATT) emerged as the most influential predictor (100% ANN importance), underscoring the importance of interaction design and interface optimization in AI painting platforms. It is recommended that system designers implement features such as prompt suggestion modules, real-time visual feedback during image generation, and gamified reward systems (e.g., “creation achievement badges”) to enhance user engagement and emotional reinforcement.

Lastly, in light of the finding that the behavioral level did not exert a significant effect on perceived enjoyment, designers are advised not to rely solely on operational smoothness to improve user experience. Rather, an emotional sedimentation mechanism should be established to reinforce affective engagement. Practical implementations may include “My Emotional Gallery” for archiving artworks, tools for tracking emotional changes over time, and narrative logging features to facilitate the internalization and expression of emotional memories.

In summary, this study not only identifies key determinants of adolescent engagement with AI-assisted art therapy tools but also proposes multidimensional optimization strategies for educators, therapists, and interface designers. These recommendations aim to facilitate the effective integration and broader adoption of generative AI painting technologies within mental health support contexts.

### 5.3 Limitations and future directions

It is explicitly stated that the nature of cross-sectional designs limits the ability to infer causal relationships and to monitor dynamic changes in engagement or interest development over time. To address these limitations, a longitudinal follow-up design has been proposed for future research to examine the evolution of adolescents’ interest and emotional engagement in generative AI painting tools.

Specifically, a six-month follow-up study is planned to be conducted within routine art therapy workshops or school-based mental health programs. The study will consist of six structured observation points (T1–T6), incorporating monthly AI painting activities and corresponding feedback. At each time point, three categories of data will be collected:

- Questionnaire-based assessments of interest-driving mechanisms—including perceived fun (PF), perceived enjoyment (PE), perceived trust (PT), and continued use intention—will be employed to evaluate temporal dynamics.
- Open-ended emotional response logs will be collected, with participants reflecting on their affective experiences following each AI painting activity.
- Behavioral data—including real-time input, image generation frequency, and saving behaviors—will also be recorded to track cognitive adjustment and system interaction patterns.

These longitudinal data will enable the application of advanced statistical modeling techniques such as latent growth models (LGMs) or cross-lagged structural equation models (SEMs). For instance, the extent to which initial levels of perceived trust predict sustained interest—or whether increases in perceived enjoyment are associated with cumulative emotional engagement—can be statistically tested. Such analyses may reveal how short-term novelty develops into long-term emotional attachment to AI-based tools. It is believed that these proposed strategies enhance both the theoretical depth and practical applicability of future research, thereby addressing the reviewers’ concerns about methodological robustness.

The study was conducted in Hubei Province, China, where adolescents tend to exhibit relatively high digital literacy due to national AI education policies. However, their perceptions of psychological support tools, such as art therapy, may differ significantly from those held in Western contexts. For example, conventional forms of art therapy are often associated with clinical stigma, potentially reducing adolescents’ willingness to participate openly. In contrast, AI-assisted digital painting tools tend to be perceived as novel and less stigmatized, offering a more culturally acceptable avenue for emotional expression.

To enhance the cross-cultural generalizability of the findings, replication studies are recommended in Western countries. Adolescents in these settings may hold differing attitudes toward therapy, possess greater exposure to digital creative platforms, and adhere to distinct norms of emotional expression. Specifically, future studies could implement localized versions of instructional content, apply parallel experimental protocols, and perform measurement invariance tests to examine the robustness of the model across cultural groups. Additionally, qualitative comparisons of emotional narratives and usage behaviors may uncover culturally specific engagement mechanisms. This cross-cultural perspective is expected to enrich theoretical models of emotion-driven AI interaction and inform the design of culturally adaptive digital art therapy tools.

## Data Availability

The data analyzed in this study are subject to the following licenses/restrictions: the data analyzed for this paper (retrospectively) include digital interaction records from AI-driven mental health interventions. Therefore, the dataset cannot be made publicly available. The authors will take any reasonable requests for de-identified data under consideration. Requests to access these datasets should be directed to baoqian@hanyang.ac.kr.
